# Evaluation of tractography-based myelin-weighted connectivity across the lifespan

**DOI:** 10.3389/fnins.2023.1228952

**Published:** 2024-01-04

**Authors:** Sara Bosticardo, Simona Schiavi, Sabine Schaedelin, Matteo Battocchio, Muhamed Barakovic, Po-Jui Lu, Matthias Weigel, Lester Melie-Garcia, Cristina Granziera, Alessandro Daducci

**Affiliations:** ^1^Diffusion Imaging and Connectivity Estimation (DICE) Lab, Department of Computer Science, University of Verona, Verona, Italy; ^2^Translational Imaging in Neurology (ThINK), Department of Biomedical Engineering, Faculty of Medicine, University Hospital Basel, Basel, Switzerland; ^3^ASG Superconductors S.p.A., Genoa, Italy; ^4^Sherbrooke Connectivity Imaging Laboratory (SCIL), Département d’Informatique, Université de Sherbrooke, Sherbrooke, QC, Canada; ^5^Research Center for Clinical Neuroimmunology and Neuroscience Basel (RC2NB), University Hospital Basel and University of Basel, Basel, Switzerland

**Keywords:** structural connectivity, myelin network architecture, myelin weighted connectome, brain aging, tractography, microstructure informed tractography

## Abstract

**Introduction:**

Recent studies showed that the myelin of the brain changes in the life span, and demyelination contributes to the loss of brain plasticity during normal aging. Diffusion-weighted magnetic resonance imaging (dMRI) allows studying brain connectivity *in vivo* by mapping axons in white matter with tractography algorithms. However, dMRI does not provide insight into myelin; thus, *combining tractography with myelin-sensitive maps* is necessary to investigate myelin-weighted brain connectivity. Tractometry is designated for this purpose, but it suffers from some serious limitations. Our study *assessed the effectiveness* of the recently proposed Myelin Streamlines Decomposition (MySD) method in estimating *myelin-weighted connectomes* and its capacity to detect changes in myelin network architecture during the process of normal aging. This approach opens up new possibilities compared to traditional Tractometry.

**Methods:**

In a group of 85 healthy controls aged between 18 and 68 years, we estimated myelin-weighted connectomes using Tractometry and MySD, and compared their modulation with age by means of three well-known global network metrics.

**Results:**

Following the literature, our results show that myelin development continues until brain maturation (40 years old), after which degeneration begins. In particular, mean connectivity strength and efficiency show an increasing trend up to 40 years, after which the process reverses. Both Tractometry and MySD are sensitive to these changes, but MySD turned out to be more accurate.

**Conclusion:**

After regressing the known predictors, MySD results in lower residual error, indicating that MySD provides more accurate estimates of myelin-weighted connectivity than Tractometry.

## Introduction

The study of brain connectivity is pivotal to unraveling brain properties in healthy individuals as well as to facilitating early diagnosis of neurodegenerative diseases (Sporns, 2016). *Diffusion-weighted magnetic resonance imaging* (dMRI) has emerged as a powerful tool for the characterization of brain structural connectivity; dMRI is sensitive to the microscopic motion of water molecules within tissues and, exploiting this information, it allows inferring *in vivo* the macroscopic trajectories of major white-matter fiber bundles in the brain, called streamlines, using *tractography* algorithms (Basser et al., 2000; Jeurissen et al., 2019). The map of anatomical connections estimated with tractography can be conveniently summarized as a graph, called *connectome* (Sporns et al., 2005), in which nodes represent gray matter nuclei and edges correspond to the axonal fibers connecting them. The number of streamlines between anatomical regions has been extensively adopted as a proxy for the strength of connections in the connectome (Bullmore and Bassett, 2011; Jones et al., 2013; Shi and Toga, 2017; Sotiropoulos and Zalesky, 2019); however, recent studies have questioned the quantitative nature of this measure (Jones, 2010; Yeh et al., 2020; Smith et al., 2022; Zhang et al., 2022) and several alternatives have been proposed to address this limitation.

*Tractometry* is a very popular and widely used technique which attempts to infer microstructure properties of the underlying neuronal tissues by evaluating a given quantitative microstructural map in the voxels along streamline trajectories (Bells et al., 2011). This strategy has been applied also to myelin-sensitive maps, e.g., myelin water fraction (MWF), magnetization transfer ratio (MTR), myelin volume fraction (MVF), and longitudinal relaxation rate (R1), with the aim of estimating the myelin content of different bundles and thus providing a more complete characterization of brain connectivity (von Keyserlingk and Schramm, 1984; Bartzokis et al., 2010; Mohammadi et al., 2015; Cercignani et al., 2017; Mancini et al., 2018; Melie-Garcia et al., 2018; Boshkovski et al., 2021, 2022). However, despite their widespread use, Tractometry-based methods present serious drawbacks when multiple fiber bundles traverse the same voxels, since all of them would be associated with the very same scalar values estimated in those voxels. Extensions have been proposed to provide more detailed microstructure estimates along distinct directions inside each voxel, such as quantitative anisotropy (Yeh et al., 2010) and fixel-based analyses (Raffelt et al., 2017), but these approaches still cannot decouple different microstructural properties of distinct fiber bundles sharing the same direction inside a given voxel, e.g., corpus callosum. Hence, Tractometry-based methods do not offer *truly* bundle-specific estimates of the microstructural properties of distinct bundles (Schiavi et al., 2022), and this limitation is evident in the construction of the connectome.

*Microstructure informed tractography* (Daducci et al., 2016), was proposed as a possible solution to overcome these limitations and provide more veridical and biologically informative estimates of brain connectivity. The basic idea is to estimate microstructural features of white-matter fibers by fitting the whole set of streamlines reconstructed with tractography, called tractogram, to the measured MRI data and modulating their individual contributions such that they accurately explain the measurements. Different algorithms have been developed (Smith et al., 2013; Daducci et al., 2014; Pestilli et al., 2014; Smith et al., 2015; Schiavi et al., 2020; Ocampo-Pineda et al., 2021) but, despite differences between them, they are all based on dMRI; hence, they cannot provide any insight into the actual myelination of different bundles (Beaulieu, 2002; Tax et al., 2021). Recently, the *Convex Optimization Modeling for Microstructure Informed Tractography* (COMMIT) (Daducci et al., 2014) was extended to enable its use with myelin-sensitive maps and provide researchers with an effective means to study the myelination of individual bundles. However, this novel Myelin Streamline Decomposition (MySD) technique (Schiavi et al., 2022) has been tested only with few and selected anatomical bundles, and no evaluation was performed to assess its effectiveness in describing myelin-weighted global connectivity.

The study of myelination in the brain is essential due to its profound impact on neural function. Myelin acts as an insulator, significantly increasing the speed and efficiency of electrical signal transmission within the nervous system, facilitating information processing and precise neuron communication (Morell and Quarles, 1999; Sampaio-Baptista and Johansen-Berg, 2017). Myelination is crucial during early development and continues to influence learning, memory, and cognitive function throughout life (von Keyserlingk and Schramm, 1984; Bartzokis et al., 2010; Billiet et al., 2015; Cercignani et al., 2017; Faizy et al., 2020; Meissner et al., 2021; Lebel et al., 2022). Understanding myelination mechanisms and regulation provides insights into neurological conditions, including demyelinating diseases (Tozer et al., 2003; Horsfield, 2005; Cunniffe and Coles, 2019; Kamagata et al., 2019; Granziera et al., 2020; Hara et al., 2020; Boshkovski et al., 2022). Therefore, studying myelination is crucial for comprehending neural communication and its implications for human health.

The literature on the study of myelin network architecture across the lifespan is limited, and the existing methods have primarily relied on Tractometry to assess connections with myelin-sensitive maps (Lebel et al., 2012; Callaghan et al., 2014; Yeatman et al., 2014; Meissner et al., 2021). In this article, we leveraged the ability of MySD to provide bundle-specific estimates of myelination with the aim to *accurately characterize changes in the myelin network architecture over the lifespan*. In fact, existing studies on connectivity alterations during normal brain aging were *mainly* focused on dMRI and limited attention has been given to possible myelin-specific changes (Nomura et al., 1994; Sullivan et al., 2001; Salat et al., 2005; Westlye et al., 2010; Kochunov et al., 2011, 2012; Lebel et al., 2012, 2022; Yeatman et al., 2014; Ota et al., 2017; Slater et al., 2019; Buchanan et al., 2020). We evaluated the effectiveness of MySD by comparing its estimates to a classical Tractometry-based approach; we also performed our analysis on dMRI-based microstructural maps as a reference to corroborate previous findings and provide additional insights. With this study, we aim to test a viable alternative to Tractometry that overcomes its inherent limitations. Our approach combines global tractography with myelin-sensitive maps in a more robust and accurate manner, effectively addressing the shortcomings of both counting the number of streamlines and traditional Tractometry methods.

## Methods

### Subjects and MRI protocol

We performed the analysis on 85 healthy controls: 46 females (median age (IQR) [range] 32.12, (27.55; 43.77), [21.62–62.00]) and 39 males (median age (IQR) [range] 34.00, (27.39, 49.97, 18.15–69.00)). All subjects underwent MRI on a 3 T system (Prisma; Siemens Healthcare, Erlangen, Germany) with a 64-channel head and neck coil.

The acquisition protocol included: *3D FLAIR* (repetition time [TR]/echo time [TE]/inversion time [TI] = 5000/386/1800 ms, 1 mm isotropic spatial resolution); *3D MP2RAGE* (TR/TI1/TI2 = 5000/700/2500 ms, 1 mm isotropic spatial resolution); and *multi-shell dMRI* with b-values 700/1000/2000/3000 s/mm^2^ and 6/20/45/66 diffusion directions per shell, respectively, as well as 12 measurements at b-value 0 s/mm^2^ with both anterior-to-posterior and reversed phase encoding (TR/TE/pulse duration [δ]/time between pulses [Δ] = 4500/75/19/36 ms, 1.8 mm isotropic spatial resolution). Three variants of a *3D FLASH* (RF spoiled GRE) sequence were used with 1.33 mm isotropic resolution, matrix size 192 × 186 × 120, PPF = 6/8; SPF = 6/8, GRAPPA_R = 2 in each phase encoding direction: T1-weighted (TR/TE = 11/4.92 ms, alpha = 15°), Proton Density weighted (TR/TE = 25/4.92 ms, alpha = 5°), MT-weighted [TR/TE = 25/4.92 ms, alpha = 5°, Gaussian MT pulse Delta_f = 2.2KHz as in Helms et al. (2008)]. B1 maps to correct for effects of radio frequency transmit inhomogeneities on the quantitative maps were acquired employing the steady state free precession based B1-TRAP approach (Ganter et al., 2013).

### Anatomical images processing

We used MRtrix3 with FreeSurfer algorithm (Tournier et al., 2019) to segment the MP2RAGE images into five separate masks corresponding to the main tissue types in the brain (white matter, cortical gray matter, subcortical gray matter, cerebrospinal fluid, pathological tissue), which was used to guide tractography with anatomical information; using these masks, we also calculated the gray matter-white matter interface. In addition, to define the connectome nodes, we further segmented the cortical and subcortical tissues with FreeSurfer 6.0 (Fischl, 2012) into 85 regions of interest [42 per hemisphere + brainstem (Iglesias et al., 2015)], as defined in the Desikan–Killiany atlas (Desikan et al., 2006; Iglesias et al., 2015). Finally, we used the boundary-based linear registration tool implemented in FSL (Jenkinson et al., 2002) to register all previous masks to the diffusion space.

### Myelin images processing

Myelin volume fraction (MVF) maps were estimated as MVF=αMTsat , where MTsat is defined as the portion of free water saturated during a single MT pulse, and the calibration constant α was estimated based on the procedure described in Mohammadi et al. (2015). The splenium of the corpus callosum from 26 healthy subjects (mean age 27.9 ± 1.3 years) was used as region of interest and the value α = 0.2161 was obtained as the median normalization factor required to constrain the splenium g-ratio to 0.7 across subjects, as previously reported using the electron microscopy technique (von Keyserlingk and Schramm, 1984).

### Diffusion images processing

dMRI data was pre-processed to reduce artifacts from noise (Veraart et al., 2016a,b), eddy currents (Andersson and Sotiropoulos, 2016), motion and EPI distortions (Andersson et al., 2003; Smith et al., 2004) using MRtrix3 (Tournier et al., 2019) and FSL (Woolrich et al., 2009; Jenkinson et al., 2012). Images were also corrected for B1 field inhomogeneity using the N4 algorithm implemented in ANTs (Tustison et al., 2010). The Spherical Mean Technique (Devan et al., 2020) was applied to data with b-value ≤ 2,000 s/mm^2^ to estimate the intra-neurite volume fraction (INVF) map.

To reconstruct the whole brain tractograms, we followed the procedure described in Bosticardo et al. (2021). Briefly, we generated 3 million streamlines using the iFOD2 algorithm with anatomical priors (Smith et al., 2012) on the fiber orientation distributions estimated with multi-shell multi-tissue constrained spherical deconvolution (Jeurissen et al., 2014), seeding from the gray matter-white matter interface and propagating the streamlines with the backtrack option using a cut-off value of 0.05 and a maximum angle of 30°. To reduce the incidence of false positives (Campbell and Pike, 2014; Zalesky et al., 2016; Maier-Hein et al., 2017; Buchanan et al., 2020), we set the power parameter of iFOD2 to 3, as in Bosticardo et al. (2021), and we filtered the tractograms with COMMIT2 (Schiavi et al., 2020) to remove spurious connections or those that are incompatible with the measured data.

### Connectome estimation

We constructed the connectomes using the 85 regions of interest from the gray matter parcellation as *nodes* and the 3 million streamlines estimated with tractography as *edges*. To determine the *edge weights*, we applied Tractometry (Bells et al., 2011) and COMMIT (Daducci et al., 2014) to both diffusion (i.e., INVF) and myelin (i.e., MVF) scalar maps. It’s worth noting that the adaptation of COMMIT to handle MVF data is known as MySD. For calculating the edge weights with Tractometry, we employed MRtrix3 (Tournier et al., 2019) to sample the INVF and MVF maps along the streamlines. Subsequently, we computed the median of the values along the streamlines’ trajectories and calculated the mean of the weights for streamlines belonging to the same bundle, following the approach proposed by Boshkovski et al. (2021). In the case of COMMIT, we first fitted the streamlines to the INVF and MVF maps with the aim to estimate an individual weight for each streamline which corresponds to its overall contribution to the corresponding microstructural map. Then, the connectivity strength of each bundle was estimated aggregating the weights of those streamlines belonging to it, as described in Schiavi et al. (2020).

**Figure 1:**
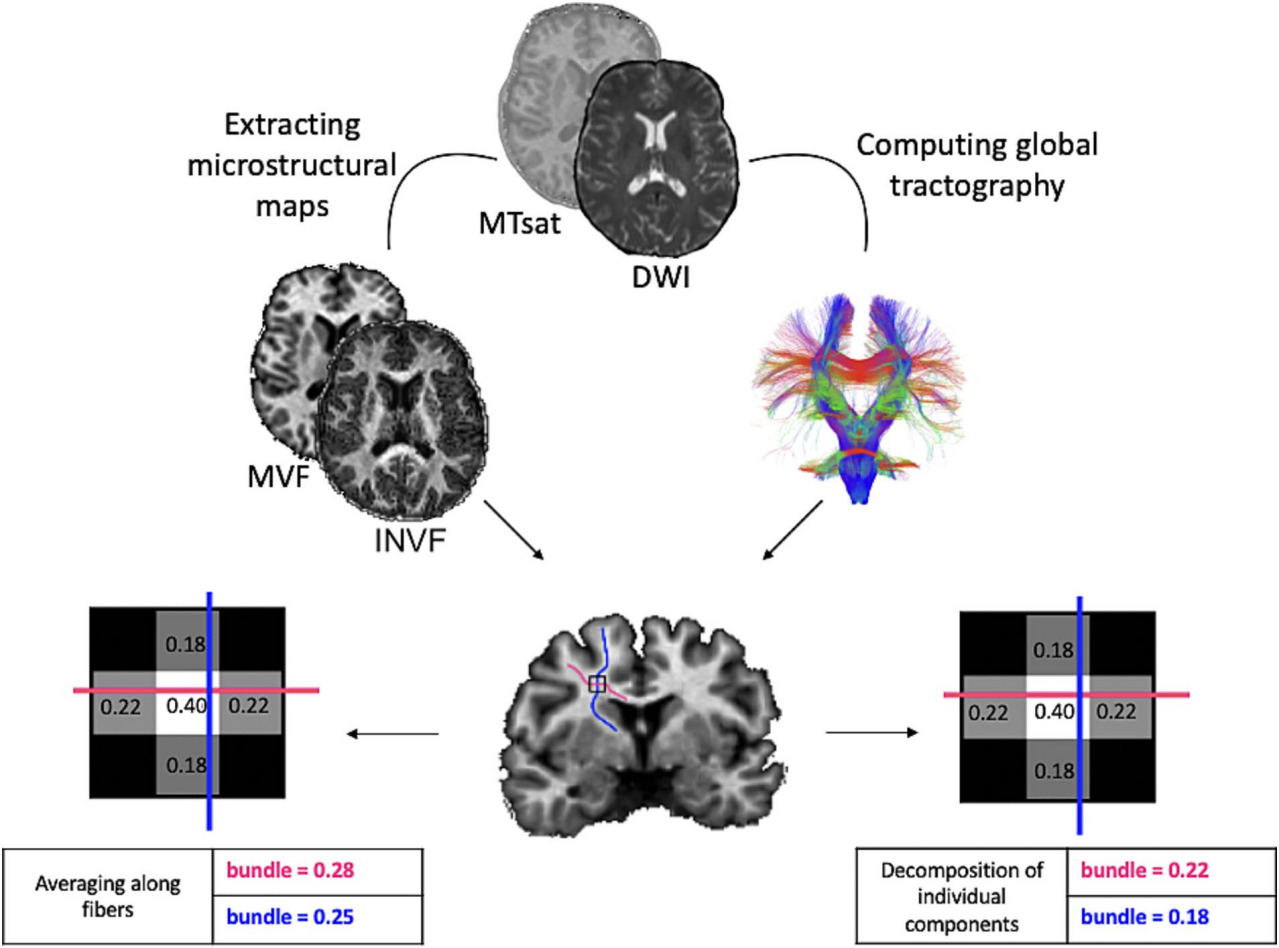
Pipeline for the construction of myelin-weighted brain graphs. We combined the myelin-sensitive MVF map with streamlines reconstructed from the diffusion image using two methods. Tractometry, which samples each streamline at *n* points to which it assigns the voxel-wise value of the underlying microstructural map and computes the average, and MySD, which solves a linear system for each voxel of the microstructural map by assigning a contribution to each streamline relative to the scalar value measured in the map. We used the same procedures to reconstruct the diffusion-weighted brain graph using INVF scalar map. MTsat, Magnetization Transfer saturation; DWI, Diffusion Weighted Image; MVF, Myelin Volume Fraction; INVF, intra-neurite volume fraction.

Figure 1 visually summarizes the main steps just described.

### Network metrics

We used the Brain Connectivity Toolbox^1^ to extract from each *weighted connectome* three network metrics that are widely used in the literature (Rubinov and Sporns, 2010; Fornito et al., 2016): *mean strength*, which corresponds to the strength of the connection between gray matter regions on average; *global efficiency*, which is the average of inverse shortest path length; *modularity*, which expresses how easily the brain connection segregates into different clusters.

Global network metrics offer a comprehensive perspective on the relationships between brain regions, surpassing what can be inferred from myelination within a white matter mask without the use of a connectome. Additionally, these network metrics highlight the delicate balance between information integration (connections between different regions) and information segregation (localized processing), which cannot be captured by white matter myelination at the voxel-wise level (Fornito et al., 2016).

### Statistical analysis

To evaluate the sensitivity of the two methods concerning myelin-weighted network changes during brain aging, we used a robust regression model, available in R^2^ (Koller and Stahel, 2011). We know from the literature that the ratio of gray matter to white matter changes throughout life (Gunning-Dixon et al., 2009; Giorgio et al., 2010; Lebel et al., 2012, 2022); since we wanted to study white-matter microstructural changes due to age, we considered the white-matter volume as independent variable in our model. Considering that gender is significantly related to brain volume, we checked the collinearity of the model using collinearity diagnostic in R (Belsley et al., 2005). Then, we tested associations between age, age^2^, and network metrics with gender and white-matter volume as independent variables. To further compare the validity of the two methods (COMMIT and Tractometry), we tested the same model in predicting the age effect using internal k-fold cross-validation (Berrar, 2018). Specifically, we randomly split the dataset into *k* = 5 sub-groups. We estimated the statistical model on *k-1* sub-groups (80% of the subjects). We tested this model on the remaining 20% of the subjects to estimate the mean square errors (MSE). The MSE indicates the mean quadratic discrepancy between the observed and the estimated data. We repeated the steps described above five times. Then, we averaged the MSEs as follows to get the cross-validation error:


$$\mathrm{CrossValidation}\;\mathrm{error}=\frac{1}{n}\overset{n}{\underset{i=1}{\sum }}MSE_{i}\text{,}$$

where *n* is the number of folds (*n* = 5 in our case).

## Results

### Myelin-weighted connectomes

**Figure 2:**
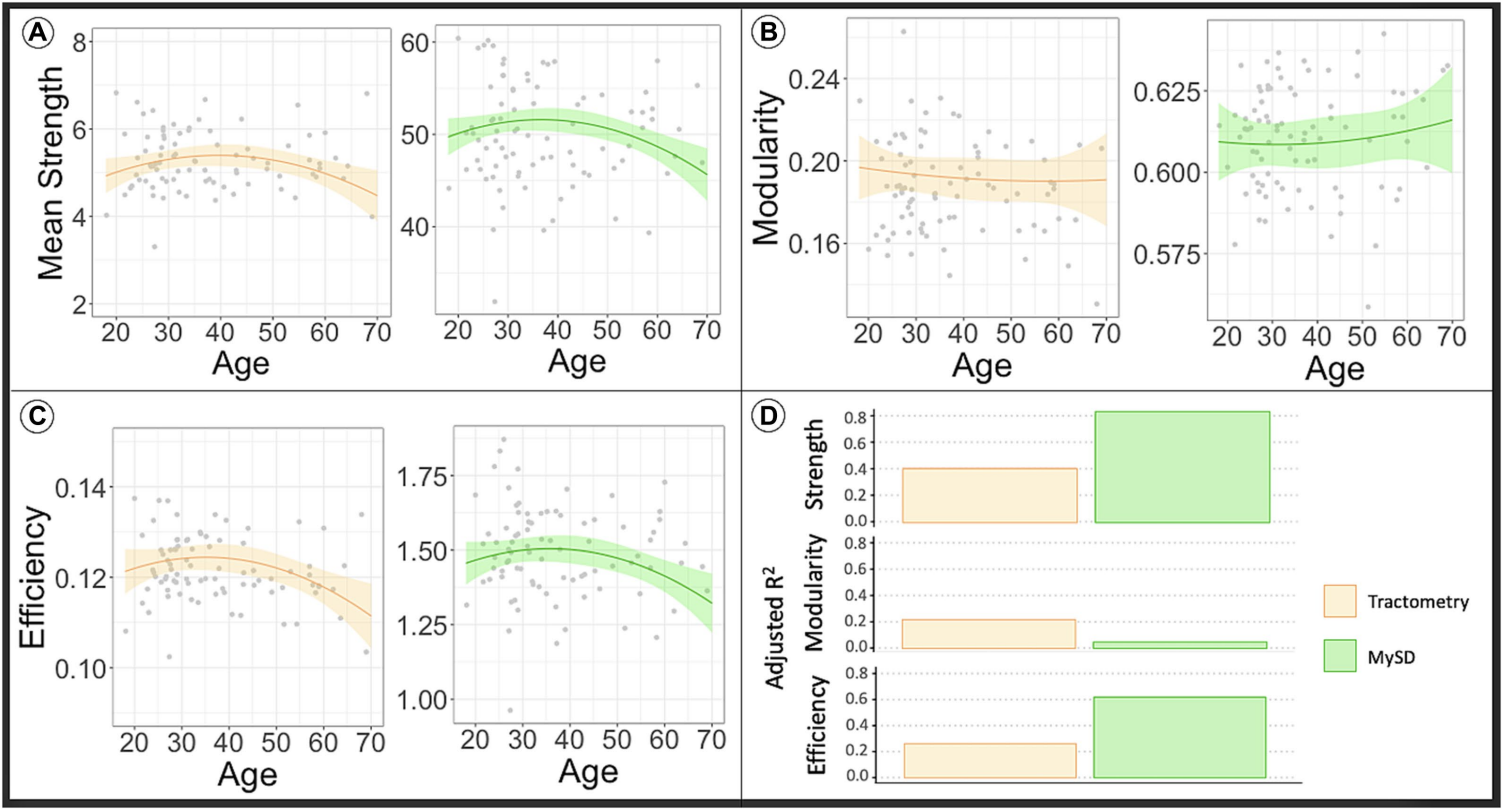
The line in the plot shows the predicted values of network metrics [**(A)** Mean strength, **(B)** modularity, **(C)** efficiency] of interest in dependence of age, while raw network metrics values are reported as gray dots. Based on the model to assess the impact of age in the global network metrics considering sex and WM volume as covariates, we predicted the global network metrics for age in range 20–70. We fixed sex as males and WM volume as the average value found in our sample. In orange are reported the predicted network metrics from the model fitted on Tractometry weighted connectomes, while in green are reported the network metrics computed using MySD. In panel **(D)** we reported the plot of the adjusted R^2^ values for each model. High R^2^ indicates that a lot of variance in the data is explained by the model. As first observation we can say that myelin-weighted network metrics peak around forty in all the models. On the other hand, we see a bigger R^2^ in case of MySD indicating its capacity to offer more precise estimates of myelin-weighted connectivity in comparison to Tractometry. COMMIT, Convex Optimization Modeling for Microstructure Informed Tractography; MySD, Myelin Streamlines Decomposition; MVF, Myelin Volume Fraction.

In Figure 2, the orange line in the plots displays the predicted network metrics in relation to age derived from the connectome obtained using the Tractometry-based approach, while the gray points depict the raw values of network metrics. On the other hand, the green line represents the predicted network metrics with respect to age extracted from connectomes generated using the MySD method, along with the raw values depicted as gray dots. The observations from these plots indicate that the global mean connectivity strength and efficiency (panel A and panel C, respectively), computed from the myelin-weighted connectomes produced by both modalities, exhibit a curve resembling an asymmetric inverted U-shape as a function of age. These curves reach their peak around the age 40 years, which aligns with previous experimental findings using different myelin sensitive values (Yeatman et al., 2014).

The upper section of Table 1 presents the outcomes of statistical analyses conducted to compare the effectiveness of the methodologies in capturing myelin changes at different age stages. Consistent with the plots depicted in Figure 2, the results demonstrate that both Tractometry (top left of the table) and MySD (top right of the table) are sensitive to alterations in global efficiency and mean connectivity strength of myelin-weighted networks. However, MySD proves to be more precise in identifying myelin changes compared to Tractometry, as evidenced by the model’s superior goodness of fit. As depicted in Figure 2D, the estimated model based on MySD data exhibits goodness of fit at least twice as high as the one obtained with Tractometry (p_age-square_ = 0.031, R^2^ = 0.606; p_age-square_ = 0.035, R^2^ = 0.257, respectively for global efficiency and p_age_ = 0.023, p_age-square_ = 0.009, R^2^ = 0.781; p_age_ = 0.022, p_age-square_ = 0.015, R^2^ = 0.392, respectively for mean strength). Additionally, our findings highlight that white-matter volume is necessary for explaining the observed data (*p* < 0.05), and the collinearity with sex does not impact the results (variance inflation factor < 2). Furthermore, the mean squared errors (MSEs) obtained from the five-fold cross-validation test indicate that the Tractometry-based approach yields MSE values twice as high as those computed with MySD (MSE = 0.886, MSE = 0.441, respectively for efficiency and MSE = 0.822, MSE = 0.361, respectively for mean strength). Consequently, the discrepancy between the tested and predicted data using Tractometry-based approach is at least twice as large as the discrepancy when using MySD. Thus, after accounting for the known predictors through regression, MySD demonstrates a significantly lower residual error. This suggests that MySD potentially offers more precise estimations of myelin-weighted connectivity compared to Tractometry. As the age distribution in the dataset was not uniform, we repeated the analysis by splitting the sample to match the age distribution of the whole dataset in each subgroup; however, results do not change our conclusions, but for the sake of completeness we report them in the Supplementary Figure S1, Supplementary Tables S1, S2.

**Table 1:** In the upper part of the table are reported the results of the robust regression model applied to data from *myelin-weighted* connectomes using Tractometry (on the left) and MySD (on the right) between network metrics age and age^2^, accounting for sex and white-matter volume as covariates, while in the bottom part of the table are reported the results of the robust regression model applied to data from *diffusion-weighted* connectomes using Tractometry (on the left) and COMMIT (on the right) between network metrics age and age^2^, accounting for sex and white-matter volume as covariates.

MVF Tractometry	Efficiency	Modularity	Mean strength	MVF COMMIT (i.e., MySD)	Efficiency	Modularity	Mean strength
Age value of *p*	0.080	0.715	0.022*	Age value of *p*	0.071	0.760	0.023*
Age estimate	7.5e−4	-4.9e-4	8.0e-2	Age estimate	1.1e-2	−3.1e-4	3.9e−1
Age^2^ value of *p*	0.035*	0.787	0.015*	Age^2^ value of *p*	0.031*	0.673	0.009*
Age^2^ estimate	−1.1e-5	4.3e-6	−1.0e-3	Age^2^ estimate	-1.6e-4	5.0e-6	−5.4e-3
Sex value of *p*	0.349	0.155	0.780	Sex value of *p*	0.654	0.484	0.091
Sex estimate	1.8e-3	8.8e-3	−4.3e-2	Sex estimate	−1.3e-2	−3.3e-3	1.3
WM volume value of *p*	0.002*	<0.001*	<0.001*	WM volume value of *p*	<0.001*	0.037*	<0.001*
WM volume estimate	3.7e-8	−1.7e-7	5.4e-6	WM volume estimate	1.5e-6	5.9e-8	5.7e-5
R^2^	0.257	0.216	0.392	R^2^	0.606	0.035	0.781
MSE	0.886	0.905	0.765	MSE	0.441	1.029	0.251

INVF Tractometry	Efficiency	Modularity	Mean strength	INVF COMMIT	Efficiency	Modularity	Mean strength
Age value of *p*	< 0.001*	0.964	0.001*	Age value of *p*	< 0.001*	0.250	< 0.001*
Age estimate	6.2e-3	5.6e-5	4.2e-2	Age estimate	7.6e-2	−1.3e-3	2.6
Age^2^ value of *p*	< 0.001*	0.835	0.001*	Age^2^ value of *p*	< 0.001*	0.213	< 0.001*
Age^2^ estimate	−7.0e-5	−3.0e-6	−4.8e-3	Age^2^ estimate	−8.9e-4	1.7e-5	−3.1e-2
Sex value of *p*	0.849	0.183	0.757	Sex value of *p*	0.225	0.603	0.719
Sex estimate	−1.38e-3	7.6e-3	−4.1e−1	Sex estimate	−1.0e-1	2.8e-3	9.3e-1
WM volume value of *p*	< 0.001*	< 0.001*	< 0.001*	WM volume value of *p*	<0.001*	0.507	< 0.001*
WM volume estimate	1.7e-7	-1.6e-7	1.9e-5	WM volume estimate	5.0e-6	2.1e-8	1.8e-4
R^2^	0.264	0.252	0.368	R^2^	0.635	0.017	0.741
MSE	0.822	0.826	0.747	MSE	0.361	1.127	0.255

We marked the results with significant value of *p* with asterisks.

### Diffusion-weighted connectomes

**Figure 3:**
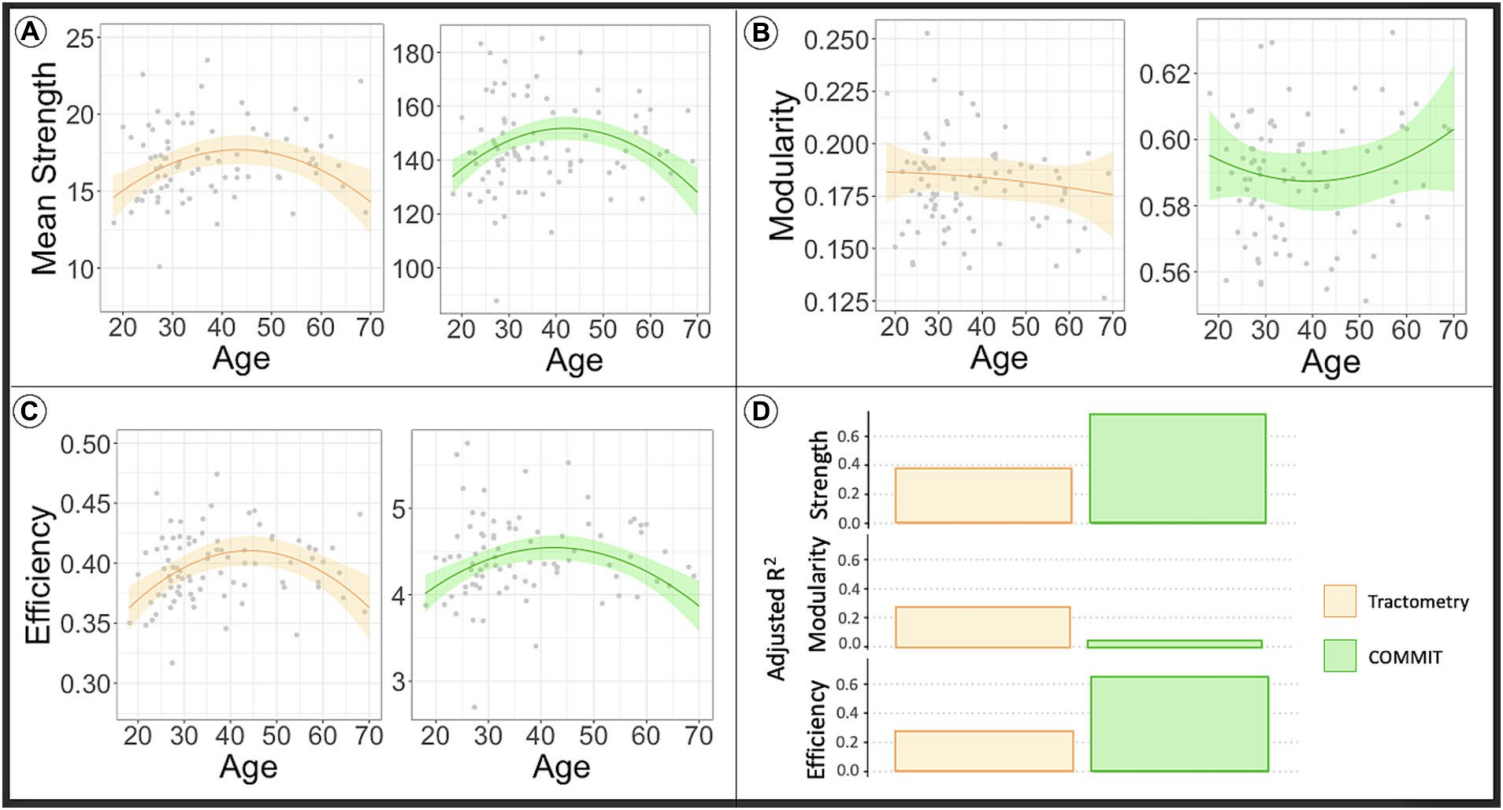
The line in the plot shows the predicted values of network metrics [**(A)** Mean strength, **(B)** modularity, **(C)** efficiency] while raw network metrics values are reported as gray dots. Based on the model to assess the impact of age in the global network metrics considering sex and WM volume as covariates, we predicted the global network metrics for age in range 20–70. We fixed sex as males and WM volume as the average value found in our sample. In orange are reported the predicted network metrics from the model fitted on Tractometry weighted connectomes, while in green are reported the network metrics computed using COMMIT. In panel **(D)** we reported the plot of the adjusted R^2^ values for each model. High R^2^ indicates that a lot of variance in the data is explained by the model. As first observation we can say that myelin-weighted network metrics peak around forty in all the models. On the other hand, we see a bigger R^2^ in case of COMMIT indicating its capacity to offer more precise estimates of diffusion-weighted connectivity in comparison to Tractometry. COMMIT, Convex Optimization Modeling for Microstructure Informed Tractography; INVF, intra-neurite volume fraction.

The lines in the plots depicted in Figure 3 show the predicted network metrics extracted from the microstructure-weighted connectomes of the INVF map using Tractometry (plots colored orange) and COMMIT (plots colored green). Raw network metric values are represented as gray points. Like the analysis using MVF map, the results show that the mean connectivity strength, and the global efficiency (panel A and panel C, respectively) extracted from the INVF-weighted connectomes calculated using both Tractometry and COMMIT, exhibit a curve resembling an inverted U-shape as a function of age that peaks around the age range of 40–50 years (Gunning-Dixon et al., 2009; Melie-Garcia et al., 2018; Slater et al., 2019; Lebel et al., 2022).

The outcomes of the statistical analysis conducted on the diffusion-weighted connectomes can be found at the bottom of Table 1. As seen above, the results show that both methods are sensitive to changes occurring in the diffusion-weighted network concerning global efficiency and mean connectivity strength. However, as depicted in Figure 3D, the values of the goodness of the fit of the model, are twice as high for analyses conducted on data calculated using COMMIT (bottom right table) as compared to data calculated using Tractometry-based approach (bottom left table) (p_age_ < 0.001, p_age-square_ < 0.001, R^2^ = 0.635; *p*_age_ < 0.001, p_age-square_ < 0.001, R^2^ = 0.264, respectively for efficiency and p_age_ < 0.001, p_age-square_ < 0.001, R^2^ = 0.741; p_age_ = 0.001, p_age-square_ = 0.001, R^2^ = 0.368, respectively for mean strength). Aligned to the previous analysis, this is reflected in the estimate of MSE (reported in Table 1), which is twice lower in the analysis conducted on connectomes using COMMIT compared to Tractometry (MSE = 0. 361, MSE = 0.822, respectively for efficiency and MSE = 0.255, MSE = 0.747, respectively for mean strength). By incorporating the known predictors through regression, COMMIT demonstrates a significantly reduced residual error, thereby affirming its capacity to offer more precise estimates of diffusion-weighted connectivity in comparison to Tractometry.

Lastly, it is important to note that when applying the statistical model to modularity computed from MySD connectomes, we observed the lowest R^2^, indicating a lot of variability not explained by the predictors used in the model. In contrast, the statistical model with modularity calculated on Tractometry connectomes is significant (R^2^ = 0.216 and R^2^ = 0.252, respectively, for MVF and INVF). In this case, the differences in the white-matter volume (*p* < 0.001) explain differences in network modularity, while age does not appear to have a significant impact on this network metric.

## Discussion

In this work, we exploited the application of MySD, a new and promising COMMIT-based method, to study changes in *global brain network properties* across the age span. We compared MySD to Tractometry (Bells et al., 2011), a commonly used method that integrates axonal and myelin properties with diffusion-based tractography to investigate myelin-weighted connectomes.

Our results show that the changes occurring in myelin network architecture due to aging have critical effects on network connection strength and efficiency (Figures 2A,C; Table 1). Specifically, we found that efficiency and mean strength extracted from myelin-weighted connectomes reach their highest point of development around 40 years of age; after this peak, the natural degeneration of axonal microstructure begins.

The literature on myelin network architecture during brain aging is not extensive (Yeatman et al., 2014; Lebel et al., 2022). Moreover, studies investigating this issue have focused on analyzing myelin relative to specific bundles or ROIs rather than globally (Gunning-Dixon et al., 2009; Bartzokis et al., 2010; Callaghan et al., 2014; Yeatman et al., 2014; Billiet et al., 2015; Melie-Garcia et al., 2018; Lebel et al., 2022). These studies show that, *as found in our results*, myelin changes follow an asymmetrical inverted U-shaped curve with a peak around 40 years of age (Yeatman et al., 2014). Thus, late-maturing brain tissues, such as myelin, are subject to retrogenesis, i.e., they are particularly vulnerable to degeneration during brain aging (Yeatman et al., 2014). Moreover, these tissues follow the reverse sequence to maturation during degeneration (Yeatman et al., 2014).

The curves shown in the plots presented in Figures 2, 3 highlight the distinction in tissue maturation between myelin-weighted connectomes and diffusion. Myelin plays a crucial role during the developmental years, which, unfortunately, we have not included in our sample. In fact, for myelin, we can see that the peak of network metrics slightly precedes the peak of the network metrics extracted from diffusion-weighted connectomes. Although our results show that changes in myelin are less striking with respect to axonal density, we showed that the MySD method identifies and quantifies myelin degeneration besides providing more reliable estimates of connectivity estimates. The obtained results are promising. The ability to measure the actual myelin volume fraction for each bundle, at a global level, is of paramount importance, especially in neurodegenerative diseases. Applying this method to patient data will provide the opportunity to examine the effects of demyelination credibly and accurately on brain structure.

In the broader context of our study, which explores the *overall architecture of the myelin-weighted connectome*, MySD outperforms traditional Tractometry-based approaches in detecting myelin network changes during normal aging. This disparity may stem from differences in their inherent definitions. Tractometry, for instance, combines microstructural maps with the reconstructed tractogram by sampling the streamlines at *n* points, with each point assigned the voxel-wise value derived from the underlying microstructural map. Subsequently, the average of these values is calculated along the specific streamline’s pathway. While such methods offer valuable macroscopic insights, they present an issue when multiple fiber populations interdigitate within a voxel. In such cases, the same value is projected to all fibers passing through that voxel, potentially introducing bias into the results. To address this concern, Boshkovski et al. proposed using the median instead of the average. This approach is highly recommended for two primary reasons: (i) the median is less influenced by outliers, and (ii) it does not assume a normal distribution of values along the bundle. For a comprehensive perspective, we also present results of Tractometry using the mean instead of the median for INVF in Supplementary Table S3, showing consistent outcomes. In contrast, COMMIT addresses this issue by deconvolving specific microstructural features for each fiber, allowing the recovery of individual streamlines’ contributions to the measured signal.

In our diffusion-based analyses, we selected the INVF map from SMT due to its stability in handling crossing fibers, a common occurrence in neuronal structures. A previous study investigating the effects of aging on network metrics used the ICVF map from Neurite Orientation Dispersion and Density (NODDI) (Buchanan et al., 2020). Both INVF and ICVF maps are sensitive to axonal density, and their values within the white matter (WM) are highly correlated (*r* = 0.87). To bolster the robustness of our study, we conducted identical analyses using the ICVF map from NODDI, as presented in Supplementary Table S4.

One limitation of this study is the age range of the subjects (18–68 years), which may not capture typical myelin network changes (Lebel et al., 2022). While our results reveal changes during brain development and myelin degeneration, the full curve’s symmetry is unclear due to the dominance of older subjects. A broader age range could provide a better understanding. Additionally, we excluded sex*age interaction from our model due to unequal age distribution between males and females.

We know from previous studies that the ratio of gray matter to white matter changes with aging (Westlye et al., 2010; Lebel et al., 2022). Global network metrics are closely dependent on white matter volume. Smaller volumes of white matter, regardless of the cause, lead to (1) tracking problems for tractography which likely reconstructs fewer streamlines and, consequently, to (2) lower values of global efficiency and mean connectivity strength of the final connectomes. For this reason, we used white matter volume as a possible confounder in the analyses.

The primary aim of this study was to evaluate the effectiveness and robustness of the MySD method in generating *myelin-weighted global connectomes*, an application not previously explored in clinical settings. To accomplish this, we chose a relatively straightforward context, namely aging, where previous research has well-demonstrated the sensitivity of diffusion methods. Therefore, our specific emphasis on myelin in this study adds a level of detail that might otherwise be overlooked.

Nonetheless, we firmly believe that highlighting MySD’s sensitivity to changes in the myelin structure of the network could significantly impact the study of demyelinating pathologies. In cases where diffusion alone may not be sufficient to detect this phenomenon, this emphasis on MySD could prove crucial. Furthermore, in future investigations, the incorporation of longitudinal data, along with the examination of local changes in myelin-weighted structural connectivity, has the potential to substantially enhance the study’s impact.

To conclude, this study underscores the importance of considering age’s role in brain connectivity research, emphasizing its non-linear nature. Different age groups exhibit unique connectivity patterns, necessitating non-linear age models and age-specific investigations for accurate connectivity estimates. Proper age adjustment in analyses ensures more reliable and meaningful interpretations of brain connectivity results.

## Conclusion

In this study, we showcased MySD’s robustness and sensitivity to myelin network changes in normal brain aging, highlighting its accuracy and capability to overcome Tractometry limitations. Applying this approach to neurodegenerative diseases could offer valuable insights into demyelination effects.

## Data availability statement

The data analyzed in this study is subject to the following licenses/restrictions: the processed data supporting the conclusions of this article will be made available by the authors, without undue reservation. The code for the MySD method used in this study is available at https://github.com/daducci/COMMIT/wiki/Bundle-myelin-fraction-%28BMF%29-mapping-with-MySD. Additionally, we provide the scripts used to generate the findings of this study on a dedicated WIKI page in the GITHUB repository of COMMIT (https://github.com/daducci/COMMIT/wiki). Requests to access these datasets should be directed to CG, cristina.granziera@unibas.ch.

## Ethics statement

The studies involving humans were approved by the ethical review committee of the University Hospital Basel (IRB of Northwest Switzerland). The studies were conducted in accordance with the local legislation and institutional requirements. The participants provided their written informed consent to participate in this study.

## Author contributions

SB, SiS, AD, and CG: conception and design of the study. SB, SiS, SaS, P-JL, MuB, MaB, MW, and LM-G: analysis of data. P-JL, MuB, and MW: data acquisition. SB, SiS, SaS, AD, and CG drafting the manuscript. SB, SiS, SaS, P-JL, MuB, MaB, MW, LM-G, AD, and CG revised the final manuscript. All authors contributed to the article and approved the submitted version.

## Footnotes

^1^ https://github.com/aestrivex/bctpy

^2^ https://www.r-project.org
