# Supplementary material for: Evaluation of tractography-based myelin-weighted connectivity across the lifespan
Source: Front Neurosci. 2024 Jan 4;17:1228952. doi: 10.3389/fnins.2023.1228952 (PMC10794573; doi:10.3389/fnins.2023.1228952)
Supplement: Supplementary file 1 [file Data_Sheet_1.docx]

Supplementary Material

**Evaluation of tractography-based myelin-weighted connectivity across the lifespan**

**Sara Bosticardo^*^, Simona Schiavi, Sabine Schaedelin, Matteo Battocchio, Muhamed Barakovic, Po-Jui Lu, Matthias Weigel, Lester Melie-Garcia, Cristina Granziera, Alessandro Daducci**

*** Correspondence:**Corresponding Author
[sara.bosticardo@univr.it](mailto:email@uni.edu)

# Supplementary Figures and Tables

## Supplementary Figures


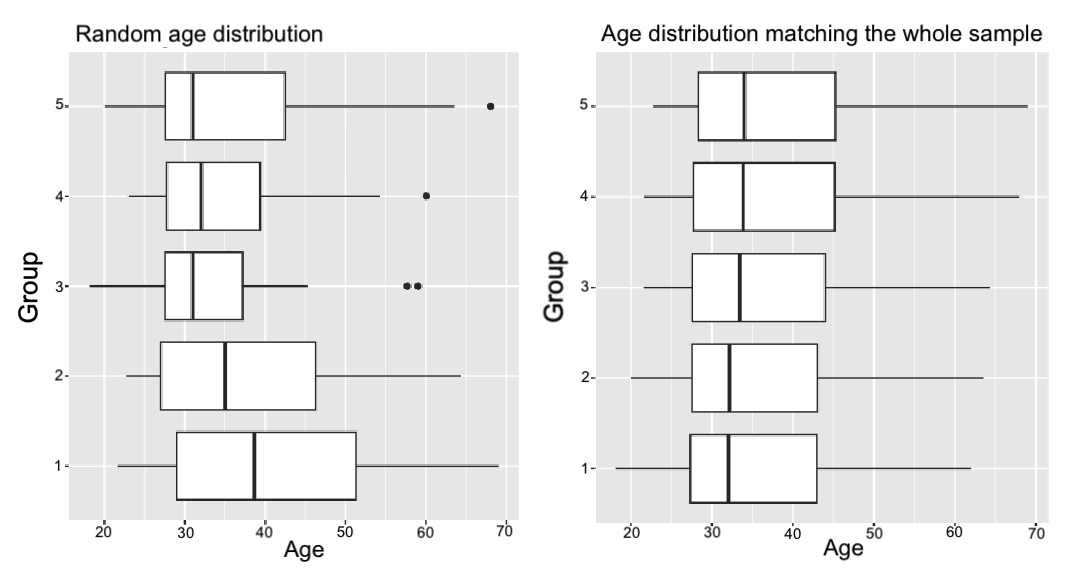


**Supplementary Figure 1.** We randomly divided the dataset into five sub-groups to calculate the mean square errors (MSEs). In the left plot, we reported the boxplot of the age distribution for each sub-group used in the k-fold cross-validation, while in the right plot, we reported the boxplot of the age distribution of each sub-group after matching it to the age distribution of the whole sample to repeat the analysis.

## Supplementary Tables

| ***Myelin Volume Fraction weighted connectomes*** | Network metric | MSE before sorting | MSE after sorting |
| --- | --- | --- | --- |
| MVF Tractometry | Global Efficiency | 0.886 | 0.890 |
|  | Mean Connectivity Strength | 0.765 | 0.723 |
| MVF COMMIT (i.e. MySD) | Global Efficiency | 0.441 | 0.415 |
|  | Mean Connectivity Strength | 0.251 | 0.237 |

**Supplementary Table 1.** In the table are reported the mean square errors obtained from the k-fold cross-validation analysis for network metrics extracted from myelin-weighted connectomes computed using MVF-Tractometry, at the top of the table, and MVF COMMIT (or MySD), at the bottom of the table, dividing the subgroups randomly (column MSE before sorting) and by matching the age range of the whole sample in each sub-group (column MSE after sorting).

| ***Intra axonal Volume Fraction weighted connectomes*** | Network metric | MSE before sorting | MSE after sorting |
| --- | --- | --- | --- |
| INVF Tractometry | Global Efficiency | 0.886 | 0.875 |
|  | Mean Connectivity Strength | 0.765 | 0.729 |
| INVF COMMIT | Global Efficiency | 0.361 | 0.373 |
|  | Mean Connectivity Strength | 0.255 | 0.272 |

**Supplementary Table 2.** In the table are reported the mean square errors obtained from the k-fold cross-validation analysis for network metrics extracted from diffusion-weighted connectomes computed using INVF-Tractometry, at the top of the table, and INVF COMMIT, at the bottom of the table, dividing the subgroups randomly (column MSE before sorting) and by matching the age range of the whole sample in each sub-group (column MSE after sorting).
**Acronyms**: intra-neurite volume fraction, INVF; Convex optimization modeling for microstructure informed tractography, COMMIT; Mean Square Error, MSE.

| **INVF**  **median *Tractometry*** | **Efficiency** | **Modularity** | **Mean Strength** | ***INVF mean Tractometry*** | **Efficiency** | **Modularity** | **Mean Strength** |
| --- | --- | --- | --- | --- | --- | --- | --- |
| **Age p-value** | < 0.001* | 0.964 | 0.001* | **Age p-value** | <0.001* | 0.779 | 0.001* |
| **Age estimate** | 6.2e-3 | 5.6e-5 | 4.2e-2 | **Age estimate** | 5.98e-03 | 3.70e-04 | 4.21e-01 |
| **Age^2^ p-value** | < 0.001* | 0.835 | 0.001* | **Age^2^ p-value** | <0.001* | 0.646 | 0.002* |
| **Age^2^ estimate** | -7.0e-5 | -3.0e-6 | -4.8e-3 | **Age^2^ estimate** | -6.71e-05 | -7.06e-06 | -4.79e-03 |
| **Sex p-value** | 0.849 | 0.183 | 0.757 | **Sex p-value** | 0.46 | 0.519 | 0.823 |
| **Sex estimate** | -1.38e-3 | 7.6e-3 | -4.1e-1 | **Sex estimate** | 4.92e-03 | 3.71e-03 | 1.25e-01 |
| **WM volume p-value** | < 0.001* | < 0.001* | < 0.001* | **WM volume p-value** | 0.006* | <0.001* | <0.001* |
| **WM volume estimate** | 1.7e-7 | -1.6e-7 | 1.9e-5 | **WM volume estimate** | 4.68e-07 | -4.98e-07 | 6.10e-05 |
| **R^2^** | 0.264 | 0.252 | 0.368 | **R^2^** | 0.214 | 0.165 | 0.288 |
| **MSE** | 0.822 | 0.826 | 0.747 | **MSE** | 0.883 | 0.922 | 0.860 |

**Supplementary Table 3.** In the table are reported the results of the robust regression model applied to data from ***diffusion-weighted*** connectomes using Tractometry with INVF from Spherical Mean Technique (SMT) computed using the average along the streamlines (on the left) and computed using the median along the streamlines (on the right). To assess the relation between global network metrics and brain aging we used a robust linear regression model accounting for sex and white-matter volume as covariates. We marked the results with significant p-value with asterisks.
**Acronyms**: intra-neurite volume fraction, INVF; White Matter, WM; Mean Square Error, MSE.

| **ICVF**  ***Tractometry*** | **Efficiency** | **Modularity** | **Mean Strength** | ***ICVF COMMIT*** | **Efficiency** | **Modularity** | **Mean Strength** |
| --- | --- | --- | --- | --- | --- | --- | --- |
| **Age p-value** | <0.001* | 0.799 | 0.002* | **Age p-value** | <0.001* | 0.561 | <0.001* |
| **Age estimate** | 5.98e-03 | 3.42e-04 | 0.418 | **Age estimate** | 7.59e-02 | -5.49e-04 | 2.88e+00 |
| **Age^2^ p-value** | <0.001* | 0.671 | 0.003* | **Age^2^ p-value** | <0.001* | 0.517 | <0.001* |
| **Age^2^ estimate** | -6.55e-05 | -6.66e-06 | -0.005 | **Age^2^ estimate** | -8.61e-04 | 7.14e-06 | -3.32e-02 |
| **Sex p-value** | 0.269 | 0.711 | 0.699 | **Sex p-value** | 0.838 | 0.380 | 0.062 |
| **Sex estimate** | 7.11e-03 | 2.17e-03 | 0.215 | **Sex estimate** | 1.66e-02 | -3.68e-03 | 5.12e+00 |
| **WM volume p-value** | 0.011* | <0.001* | <0.001* | **WM volume p-value** | <0.001* | 0.073 | <0.001* |
| **WM volume estimate** | 4.09e-07 | -5.09e-07 | 5.82e-05 | **WM volume estimate** | 1.76e-05 | 1.83e-07 | 6.88e-04 |
| **R^2^** | 0.246 | 0.181 | 0.285 | **R^2^** | 0.593 | 0.008 | 0.714 |
| **MSE** | 0.864 | 0.907 | 0.866 | **MSE** | 0.446 | 1.138 | 0.344 |

**Supplementary Table 4.** In the table are reported the results of the robust regression model applied to data from ***diffusion-weighted*** connectomes using Tractometry with ICVF from Neurite Orientation Dispersion and Density Imaging (NODDI) computed using the median along the streamlines (on the left), and the connectomes computed using COMMIT with ICVF (on the right). To assess the relation between global network metrics and brain aging we used a robust linear regression model accounting for sex and white-matter volume as covariates. We marked the results with significant p-value with asterisks.
**Acronyms**: intra-cellular volume fraction, ICVF; Convex Optimization Modeling for Microstructure Informed Tractography, COMMIT; White Matter, WM; Mean Square Error, MSE.
